# Supplementary material for: Genetically encoded cell-death indicators (GEDI) to detect an early irreversible commitment to neurodegeneration
Source: Nat Commun. 2021 Sep 6;12:5284. doi: 10.1038/s41467-021-25549-9 (PMC8421388; doi:10.1038/s41467-021-25549-9)
Supplement: Supplementary file 1 — Supplementary Information [file 41467_2021_25549_MOESM1_ESM.pdf]

Page #	Supplementary Material Contents
2	**Supplementary Figure 1.** The GEDI biosensor acutely detects neuronal death and its fluorescence is dependent on extracellular Ca2+
4	**Supplementary Figure 2.** GEDI signal correlates with but often precedes other cell death indicators.
6	**Supplementary Figure 3.** GEDI signal precedes Caspase3/7 apoptotic pathway signal indicators.
8	**Supplementary Figure 4.** GEDI signal translates across experiments and time points and represents a terminal death signal
11	**Supplementary Figure 5.** GEDI reports cell death in HEK293 cells.
13	**Supplementary Figure 6.** Higher GEDI expression can give larger separation between live and dead GEDI ratios.
15	**Supplementary Figure 7.** GCaMP6f signal increases after field stimulation, but GC150-P2a-mApple signal does not.
17	**Supplementary Figure 8.** Multiplexed, high content, 4D longitudinal imaging of zebrafish larvae.
19	**Supplementary Figure 9.** Acute detection of death in live zebrafish using GCaMP7
21	**Supplementary Figure 10.** GCaMP activity correlates with PhiPhiLux signal in NTR-ablated MNs.
23	**Supplementary Figure 11.** Tricaine inhibits activity-related GCaMP activity in zebrafish larvae MNs.
25	**Supplementary Figure 12.** Death-associated increase in GCaMP signal after MTZ exposure requires NTR expression and is independent of tricaine.
27	**Supplementary Table 1.** Longitudinal time lapse imaging experiments with rodent primary neurons expressing GEDI.
29	**Supplementary Table 2.** Zebrafish lines used in study.
30	**Supplementary Table 3.** Expression Constructs used in study.
31	**Supplementary References**

A

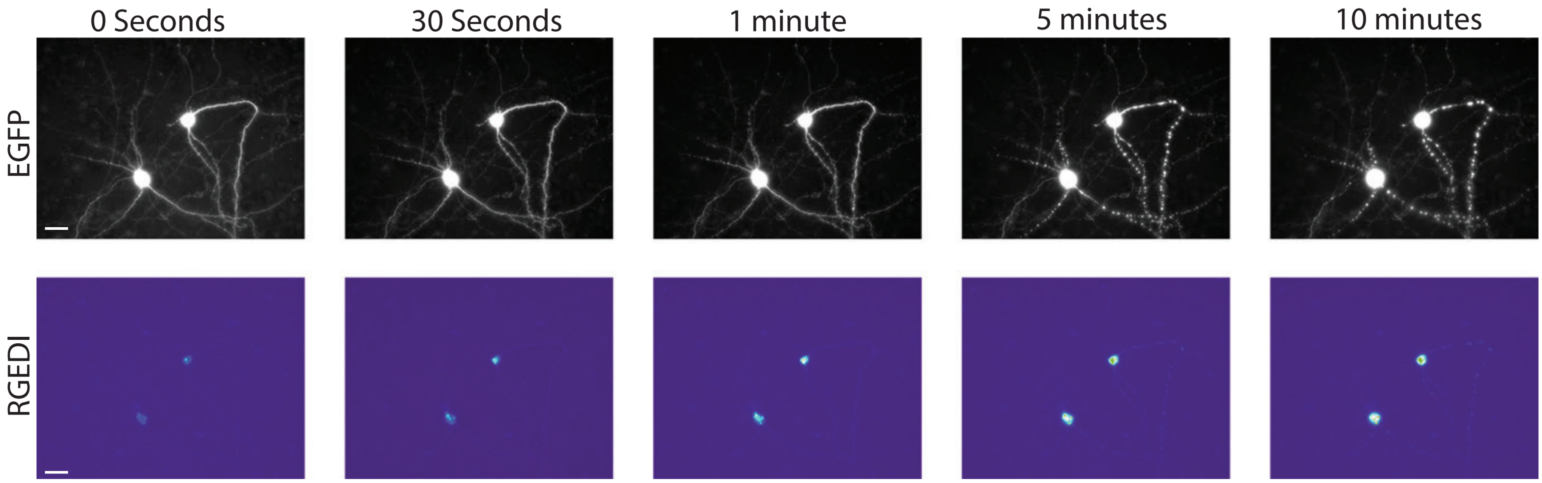
0 Seconds
30 Seconds
1 minute
5 minutes
10 minutes
EGFP
RGEDI

B

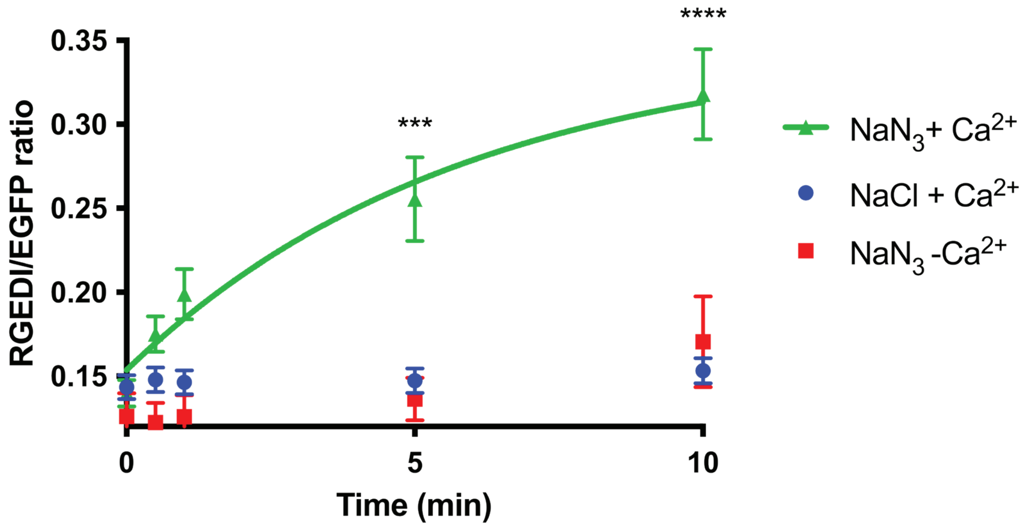
RGEDI/EGFP ratio
0.35
0.30
0.25
0.20
0.15
0
5
10
Time (min)
***
****
NaN3+ Ca2+
NaCl + Ca2+
NaN3 -Ca2+

Supplementary Figure 1. The GEDI biosensor acutely detects neuronal death and its fluorescence is dependent on extracellular Ca^{2+}

A) The GFP morphology (top) and RGEDI signal (bottom) from a time course of a rat cortical primary neurons transfected with hSyn1:RGEDI-P2a-EGFP in PBS immediately after exposure (0 seconds), or 30 seconds, 1 minute, 5 minutes, and 10 minutes after exposure to NaN_3. B) Quantification of the RGEDI/EGFP ratio during time courses of neurons treated with NaCl (control, blue circles, n=6), NaN_3 (green triangles, n=16), or NaN_3 in calcium free PBS after two washes (red squares n=11). ANOVA Tukey multiple comparisons ***= $p<0.001$, ****= $p<0.0001$. Scale = 50μm. Line represents non-linear fit using plateau followed by one phase association least squares fit (Prism 8) of NaN_3 with Ca^{2+}, R^2=0.53, τ= 5.8 minutes. NaCl and NaN_3 in calcium free PBS time courses could not be fit with the same equation. Error bars show SE.

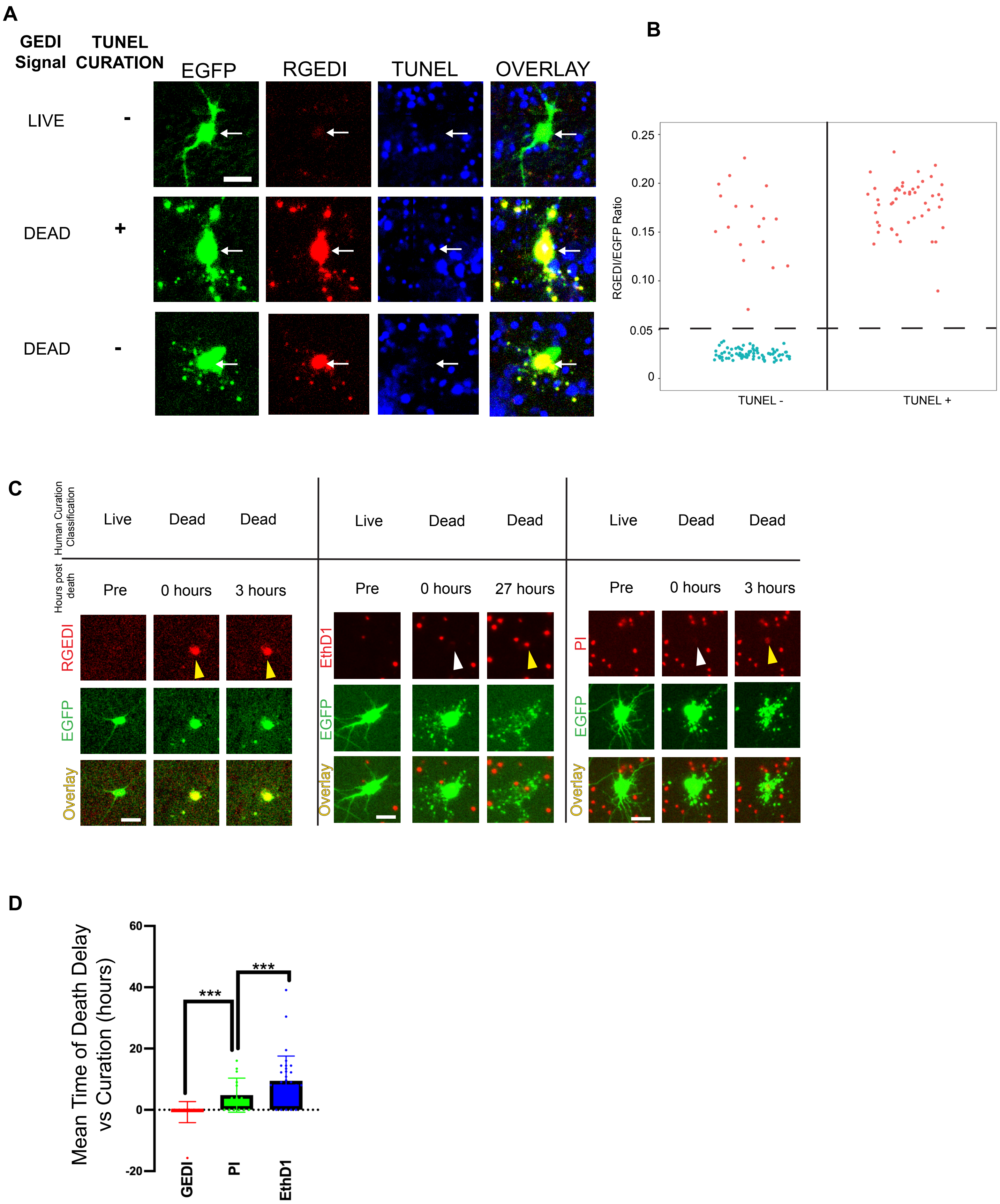
A
GEDI Signal
TUNEL CURATION
EGFP
RGEDI
TUNEL
OVERLAY
LIVE -
DEAD +
DEAD -
B
RGEDI/EGFP Ratio
0.25
0.20
0.15
0.10
0.05
0
TUNEL -
TUNEL +
C
Human Curation Classification
Hours post death
Live
Dead
Dead
Pre
0 hours
3 hours
27 hours
RGEDI
EGFP
Overlay
EthD1
PI
D
Mean Time of Death Delay vs Curation (hours)
60
40
20
0
-20
***
***
GEDI
PI
EthD1

Supplementary Figure 2. GEDI signal correlates with and often precedes other cell death indicators

A) Rat primary cortical neurons transfected hSyn1:RGEDI-P2a-EGFP and an N-terminal exon 1 fragment of a disease-associated version of the Huntingtin protein (pGW1:HTTex1Q97) then fixed and TUNEL stained. TUNEL staining was curated manually due to difficulty of automated quantification. Examples of neurons classified as live by GEDI signal and TUNEL-negative (top), dead by GEDI signal and TUNEL-positive (middle), or dead by GEDI signal and TUNEL-positive (bottom) are shown. Scale= 50µm. B) Quantification of RGEDI to EGFP ratio and classification of TUNEL-positive or TUNEL-negative by curation are shown. No live neurons were found to be TUNEL positive. C) Mouse primary cortical neurons were transfected with hSyn1:RGEDI-P2a-EGFP (left), or hSyn1:EGFP (middle and right). At 3 days in vitro (DIV), cells were treated with neural basal media (left), EthD1 (middle), or PI (right). At 6 DIV, a pretreatment image was taken (Pre), cells were exposed to 90 seconds of UV light to induce cell death, and a 0 hours image was taken, followed by additional images were taken every 3 hours. The right panel for each condition represents the first time point at which a corresponding death signal can be seen after the initial time point. Scale = 50µm. Experiment was repeated on at least 15 neurons with similar results. D) A trained human curator was shown a time series of EGFP images, and scored at what time point a neuron appeared dead by morphology. A negative time of death delay vs curation means the indicator signal preceded human curation. Bars represent mean, error bars represent SD, GEDI n=21, PI n=15, EthD1 n=35. Kruskal-Wallis ** $p<0.01$, **** $p<0.0001$.

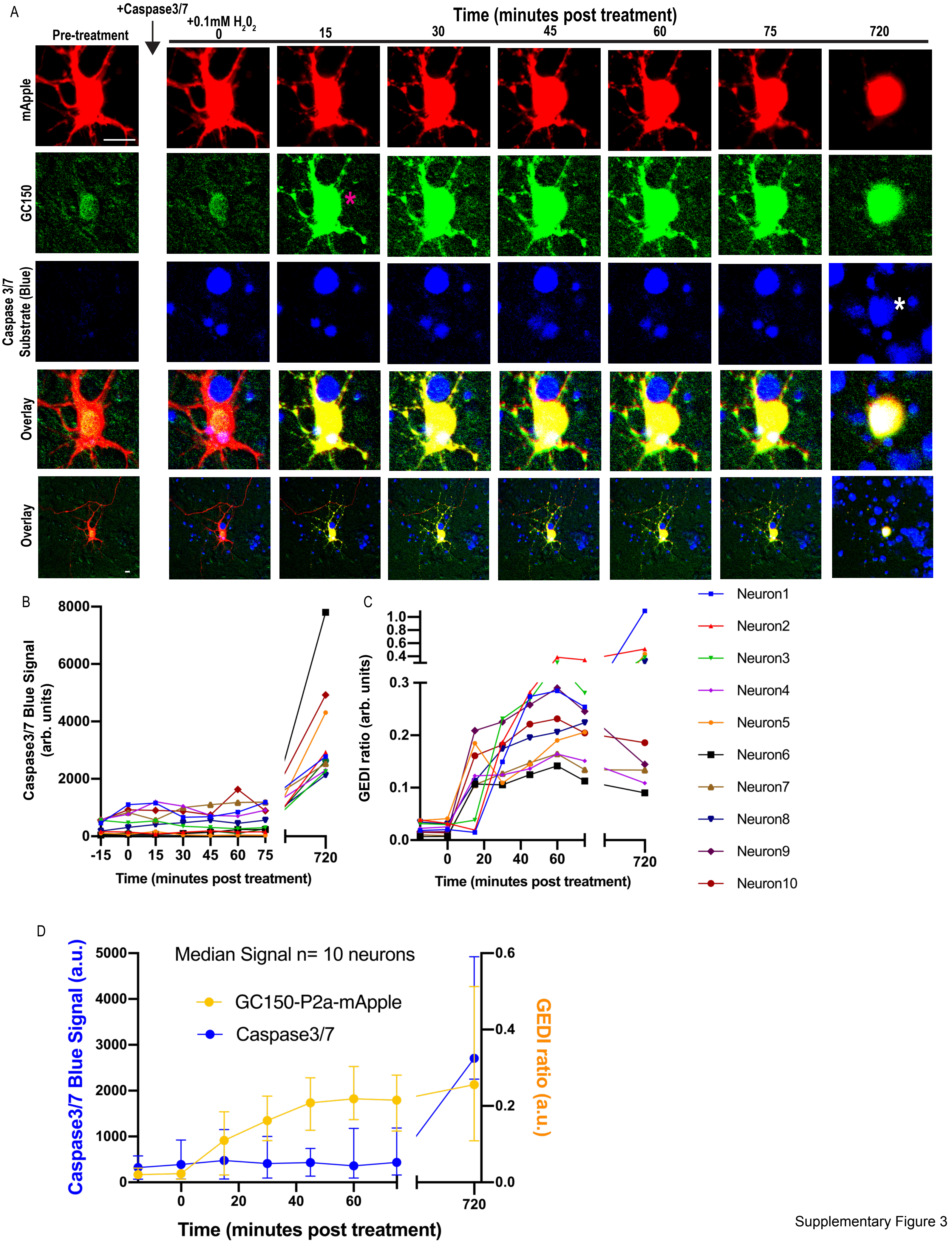

Supplementary Figure 3

Supplementary Figure 3. GEDI signal precedes Caspase3/7 apoptotic pathway signal indicators. A) Time course of GC150-P2a-mApple GEDI signal and Caspase 3/7 blue signal after induction of apoptosis with 100mM H_2O_2. Scale = 25 μm. B) Quantification of time coarse of Caspase signal from 10 neurons over 720 minutes after H_2O_2 addition. C) Quantification of GEDI ratio over 720 minutes from same 10 neurons in (B). D) Comparison of the median onset dynamics of GEDI signal and Caspase3/7 signal (+/- 95% confidence interval).

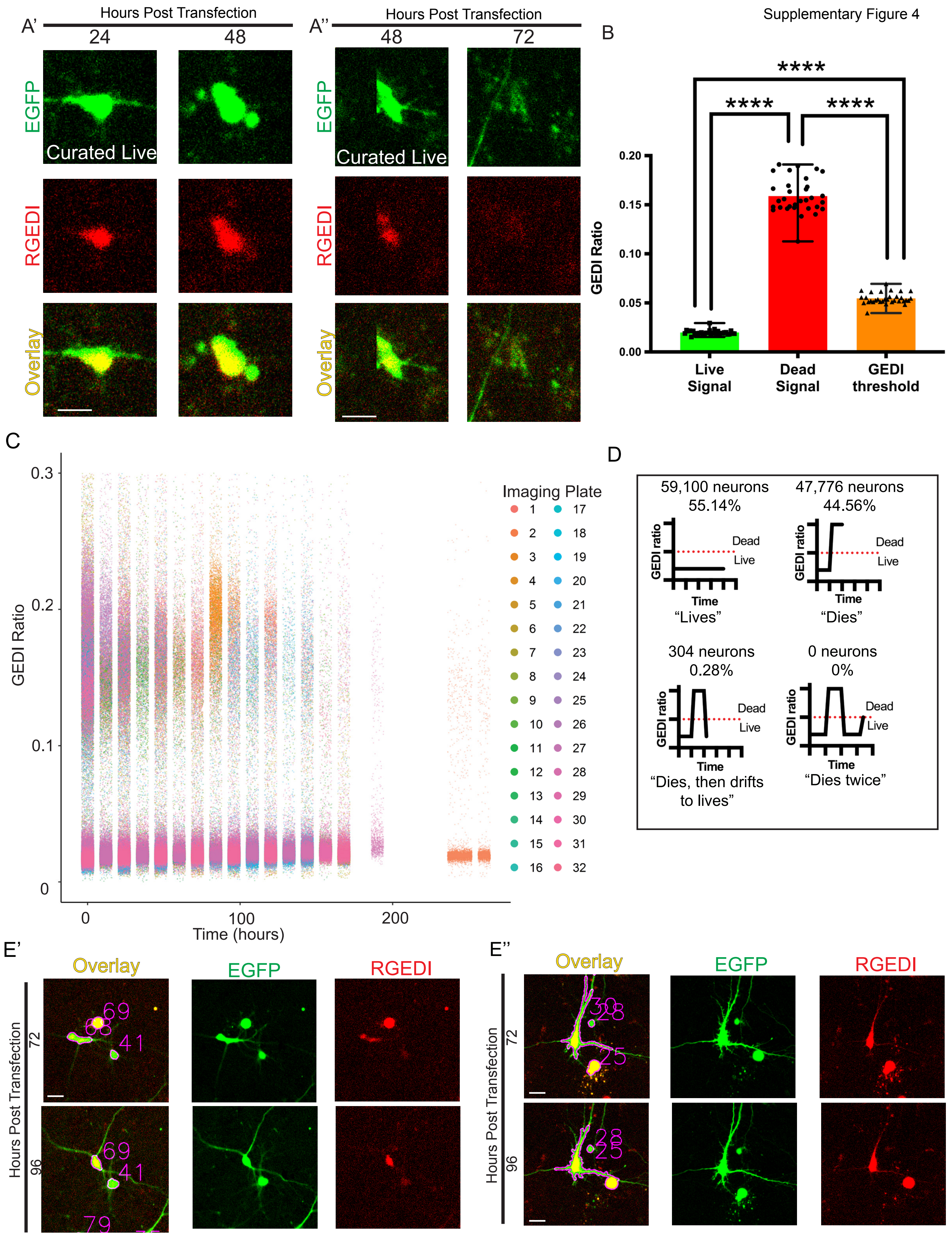
A'
Hours Post Transfection
24
48
EGFP
Curated Live
RGEDI
Overlay
A''
Hours Post Transfection
48
72
EGFP
Curated Live
RGEDI
Overlay
B
****
****
****
GEDI Ratio
0.20
0.15
0.10
0.05
0.00
Live Signal
Dead Signal
GEDI threshold
C
GEDI Ratio
0.3
0.2
0.1
0
0
100
200
Time (hours)
Imaging Plate
1 2 3 4 5 6 7 8 9 10 11 12 13 14 15 16
17 18 19 20 21 22 23 24 25 26 27 28 29 30 31 32
D
59,100 neurons
55.14%
GEDI ratio
Dead
Live
Time
"Lives"
47,776 neurons
44.56%
GEDI ratio
Dead
Live
Time
"Dies"
304 neurons
0.28%
GEDI ratio
Dead
Live
Time
"Dies, then drifts to lives"
0 neurons
0%
GEDI ratio
Dead
Live
Time
"Dies twice"
E'
Overlay
EGFP
RGEDI
Hours Post Transfection
72
96
69
68
41
79
E''
Overlay
EGFP
RGEDI
Hours Post Transfection
72
96
30
28
25

Supplementary Figure 4. GEDI signal translates across experiments and time points and represents a terminal death signal

A) Representative images of neurons curated as “live” expressing RGEDI-P2a-EGFP that have RGEDI to EGFP ratio (GEDI ratio) indicating death. EGFP channel (morphology) of neuron in (A’) at 24 hours post transfection (hpt) is difficult to classify as live or dead by eye but shows clear fragmentation of fluorescence at the next time point (48 hpt), indicative of death. Morphology of neuron in (A’’) at 48 hpt is difficult to classify as live or dead by eye but shows loss of fluorescence by next time point (72hpt), indicating death. Scale = 25 μm. B) Quantification of the mean GEDI ratio of live neurons and dead neurons and the mean GEDI threshold calculated using equation (1) from 32 independent high throughput longitudinal imaging experiments. Error bars represent range of values. ANOVA Tukey multiple comparisons **** denotes $p<0.0001$. C) GEDI ratios labeled by color over time that include 94,106 tracked neurons and 1,108,764 data points, showing stable separation of live and dead neurons across 32 experiments. D) Classification of tracked neurons based on GEDI death signal from experiments in (A) as “lives” (no death signal found), “dies” (death signal followed by disappearance of fluorescence), “dies then drifts to lives” (GEDI signal increases above GEDI threshold then decreases below threshold before loss of fluorescence), or “dies twice” (GEDI signal increases above GEDI threshold, decreases below threshold, then increases above threshold). All neurons classified as “dies then drifts to lives” show morphological features indicative of death and subsequent loss of fluorescence. E) Representative examples of tracking and segmentation errors that give “Dies then drifts to lives” artifacts. In (E’), neuron 69 (labeled with magenta numbers above and right of centroid of object) has died at 72 hpt (top) and fluorescence disappears by 96 hpt (bottom), but neuron 68 moves into the vacated position,

resulting in mislabeling of neuron 68 as neuron 69 at 72hpt. In (E’’) neuron 25 has died at 72 hpt but its debris intersects with live neuron 30 at 96hpt, causing segmentation of both neurons as neuron 25 with a lowered GEDI ratio. Scale = 25 μm. At least 12 examples of each case in E’ and E’’ were found in the dataset.

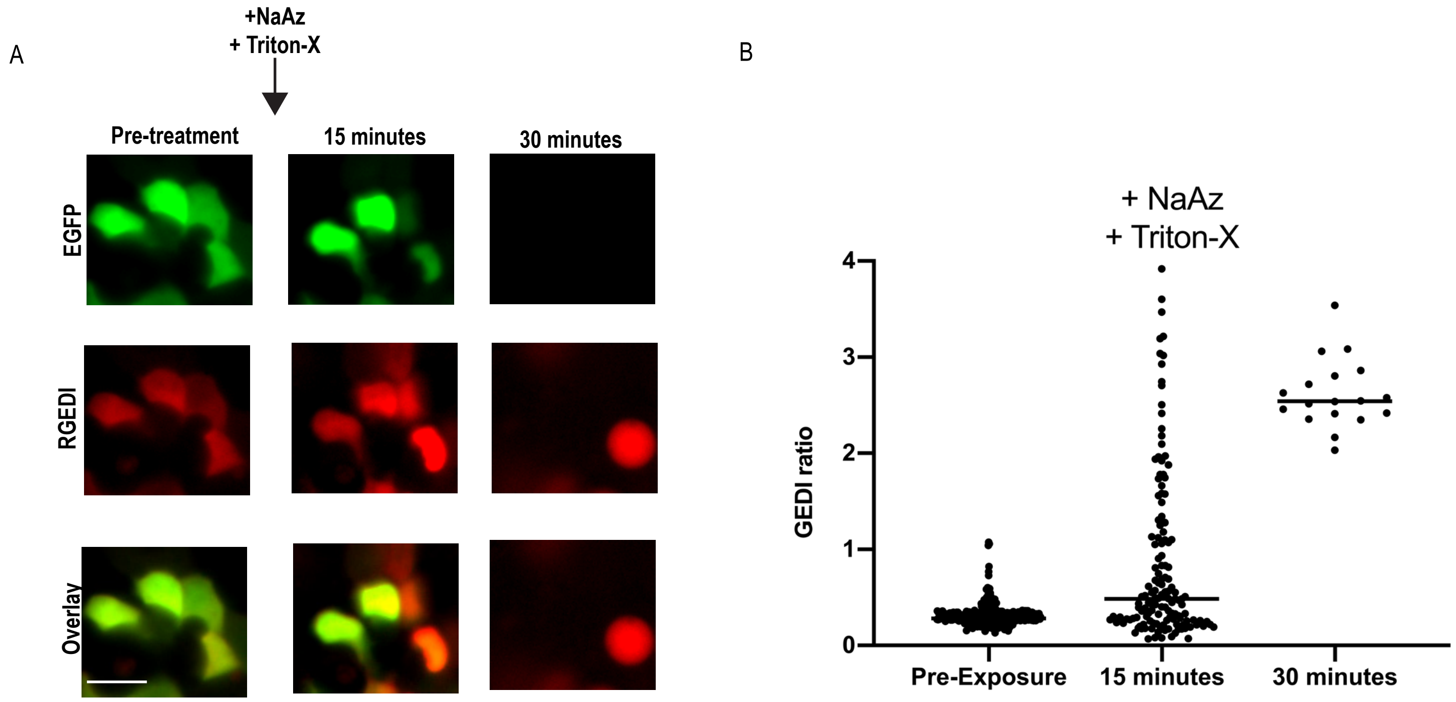

Supplementary Figure 5

Supplementary Figure 5. GEDI reports cell death in HEK293 cells.

A) Time coarse of HEK293 cells expressing RGEDI-P2a-EGFP before and after exposure to NaAz and Triton-X. Scale = 25 μm. B) Quantification of (A).

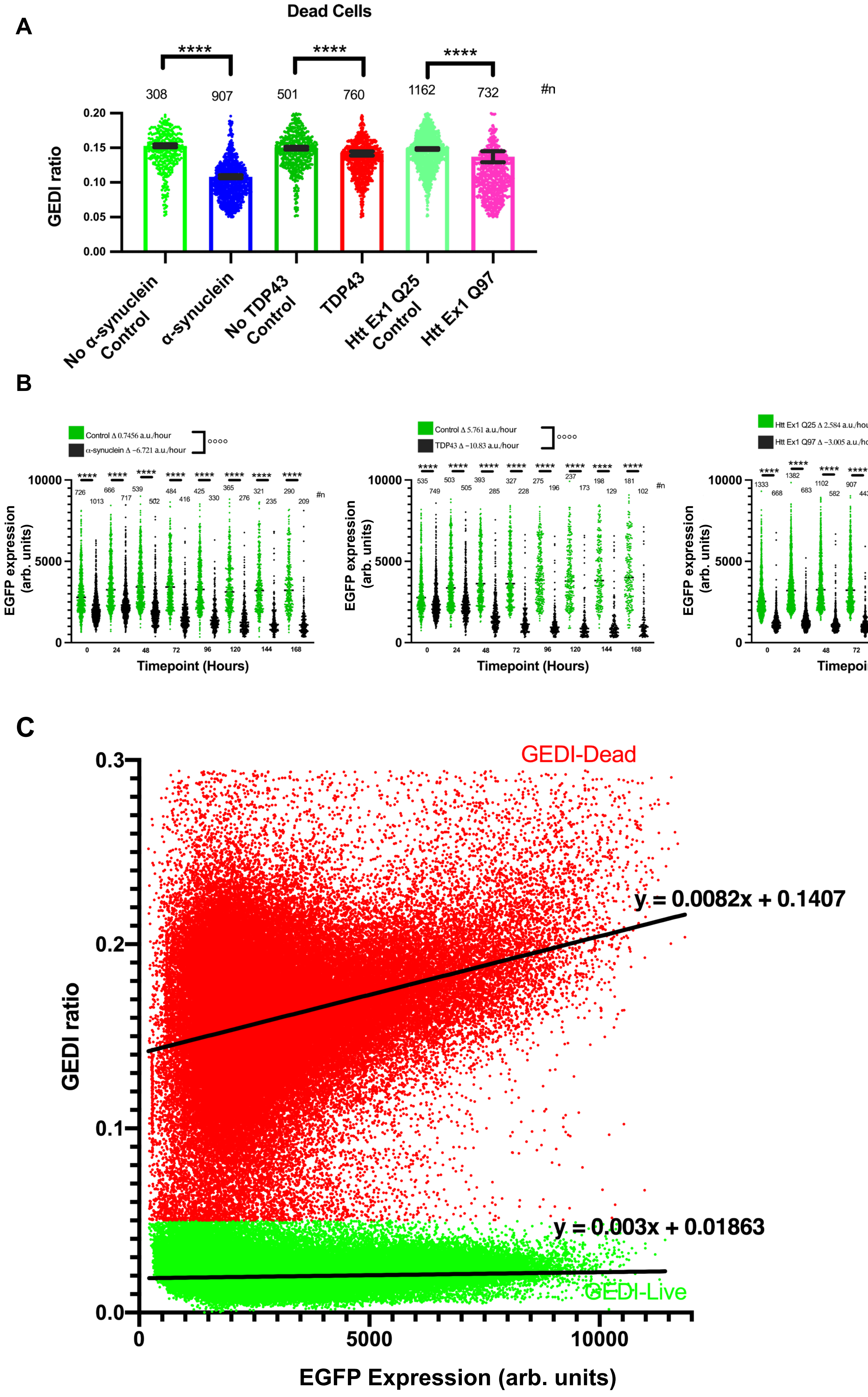
A
Dead Cells
****
****
****
308
907
501
760
1162
732
#n
GEDI ratio
0.20
0.15
0.10
0.05
0.00
No α-synuclein Control
α-synuclein
No TDP43 Control
TDP43
Htt Ex1 Q25 Control
Htt Ex1 Q97
B
Control Δ 0.7456 a.u./hour
α-synuclein Δ -6.721 a.u./hour
oooo
Control Δ 5.761 a.u./hour
TDP43 Δ −10.83 a.u./hour
oooo
Htt Ex1 Q25 Δ 2.584 a.u./hour
Htt Ex1 Q97 Δ -3.005 a.u./hour
oooo
EGFP expression (arb. units)
10000
5000
0
0
24
48
72
96
120
144
168
Timepoint (Hours)
#n
C
GEDI-Dead
y = 0.0082x + 0.1407
y = 0.003x + 0.01863
GEDI-Live
GEDI ratio
0.3
0.2
0.1
0.0
0
5000
10000
EGFP Expression (arb. units)

Supplementary Figure 6. Higher GEDI expression can give larger separation between live and dead GEDI ratios.

A) GEDI ratios of disease models have lower GEDI ratios in dead cells than their controls. Error bars show SE, ANOVA Tukey multiple comparisons **** denotes $p<0.0001$. B) EGFP expression in live cells from neurodegenerative disease models decreases over time compared to controls. Mean expression of EGFP (black bar) is reduced across all timepoints in each disease model, ANOVA Tukey multiple comparisons **** denotes $p<0.0001$, n's represent individual cells. Rate of change in EGFP expression over time (Δ) was derived from the slope of fit linear regressions, showing the each disease model with decreasing (negative Δ) and controls with increased (positive Δ) in EGFP expression over time, significantly different than controls (°°°° denotes ANCOVA $p<0.0001$). C) Expression of hSyn1:RGEDI-P2a-EGFP (EGFP intensity) within neurons plotted against their GEDI ratio showing the separation of GEDI-classified live (green) and dead (red) neurons increases slightly with increased expression using data described in Supplementary Table 1. Plotted lines represent linear regressions of dead neurons (top) and live neurons (bottom) with associated equations. Outliers were removed with robust regression and outlier removal (ROUT) in Prism using Q=1%.

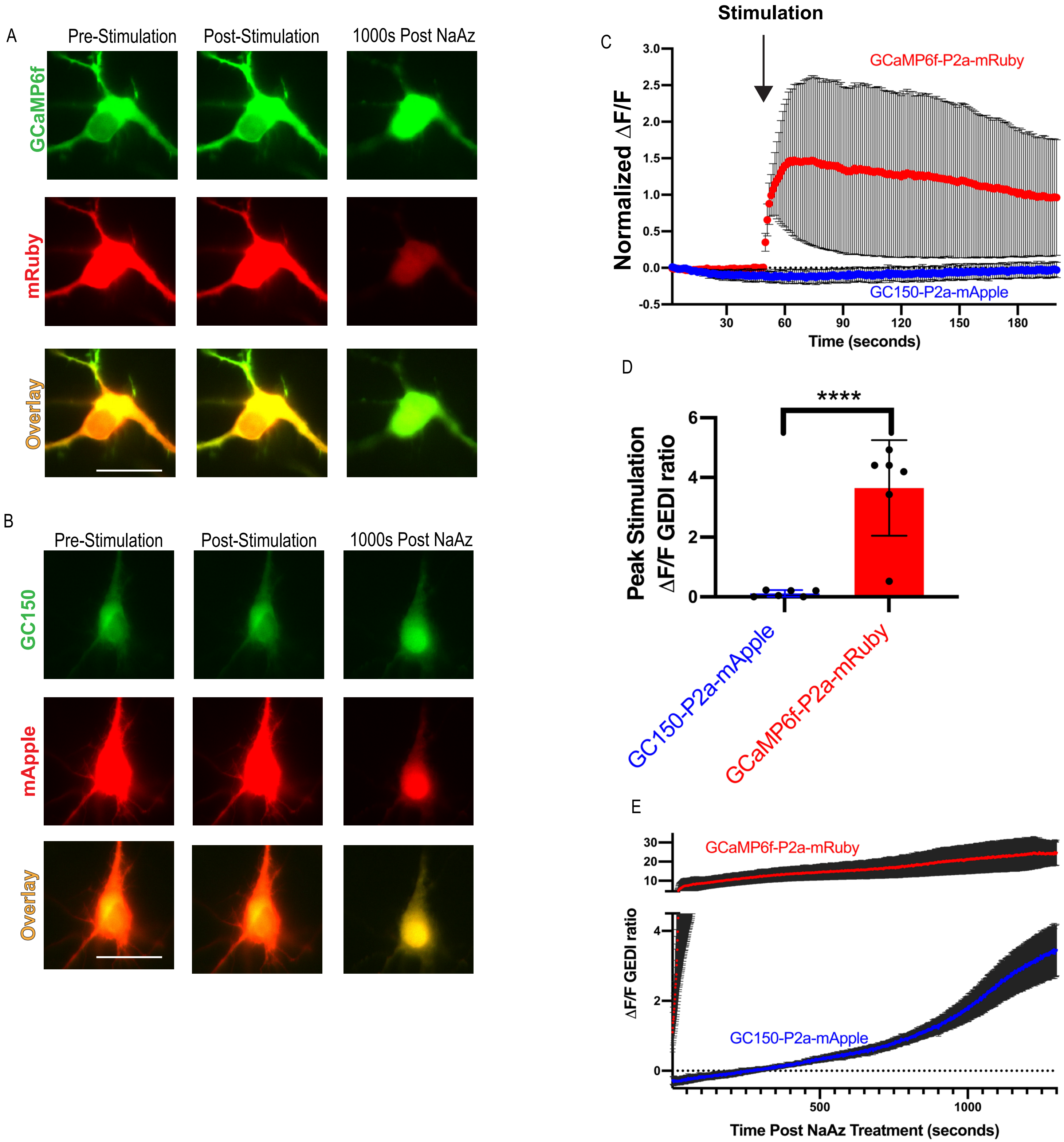
A
Pre-Stimulation
Post-Stimulation
1000s Post NaAz
GCaMP6f
mRuby
Overlay
B
Pre-Stimulation
Post-Stimulation
1000s Post NaAz
GC150
mApple
Overlay
Stimulation
C
Normalized ΔF/F
GCaMP6f-P2a-mRuby
GC150-P2a-mApple
Time (seconds)
D
****
Peak Stimulation ΔF/F GEDI ratio
GC150-P2a-mApple
GCaMP6f-P2a-mRuby
E
GCaMP6f-P2a-mRuby
ΔF/F GEDI ratio
GC150-P2a-mApple
Time Post NaAz Treatment (seconds)

Supplementary Figure 7. GCaMP6f signal increases after field stimulation, but GC150-P2a-mApple signal does not.

A) Time course imaging of GCaMP6f-P2a-mRuby signal before and after a 3 s, 30 Hz field stimulation, and after NaN_3 (10%) application. Scale= 25µm. B) Time course imaging of GC150-P2a-mApple signal before and after a 3 s, 30 Hz field stimulation, and after NaN_3 application. Scale= 25µm. C) Quantification of normalized mean ΔF/F from GCaMP6f-P2a-mRuby (GCaMP6f/mRuby ratio, n=5 coverslips, 1 neuron/coverslip) and GC150-P2a-mApple (GC150/mApple ratio, n=6 coverslips, 1 neuron/coverslip) signal after field stimulation. Error bars represent SEM. D) Peak stimulation change in GEDI ratio (**** denotes p= 0.0003 by two-tailed, unpaired T-Test). Error bars indicate +/-SEM). E) Quantification of normalized mean GCaMP6f-P2a-mApple (GCaMP6f/mRuby ratio, n=5 coverslips, 1 neuron/coverslip) and GC150-P2a-mApple (GC150/mApple ratio, n=6 coverslips, 1 neuron/coverslip) signal after NaN_3 application. Error bars represent SEM.

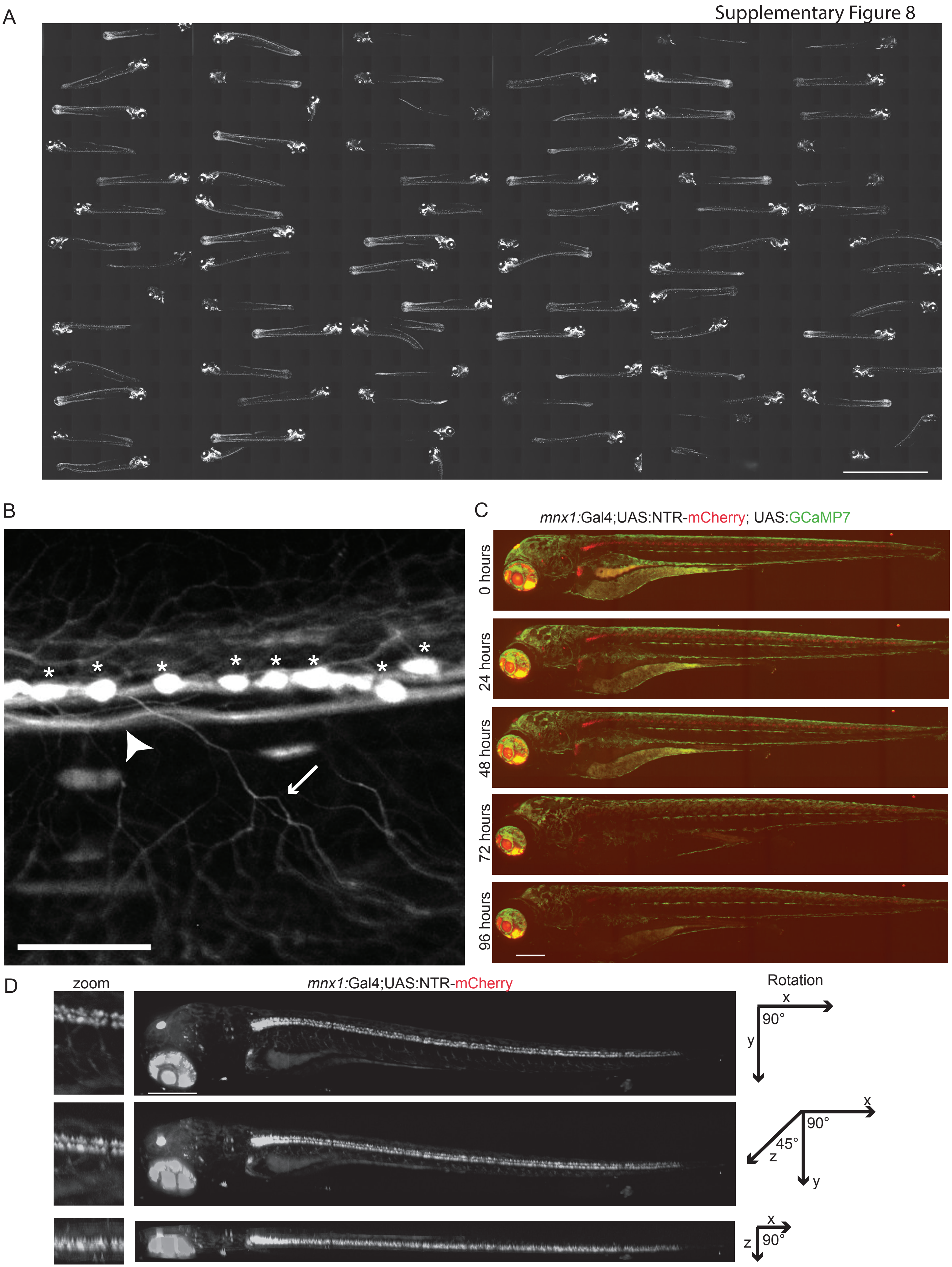
Supplementary Figure 8
A
B
C
mnx1:Gal4;UAS:NTR-mCherry; UAS:GCaMP7
0 hours
24 hours
48 hours
72 hours
96 hours
D
zoom
mnx1:Gal4;UAS:NTR-mCherry
Rotation
x
y
90°
z
45°

Supplementary Figure 8. Multiplexed, high content, 4D longitudinal imaging of zebrafish larvae. A) A representative plate of 84 live Tg:1097-Gal4; UAS:NTR-mCherry, 72 hpf zebrafish larvae immobilized in a single imaging plate imaged together, one larva per well, and montaged into a single image. Scale = 6mm. B) Representative imaging resolution of peripheral neuron somas (asterisks), axonal tract (arrowhead), and their neurites (arrow) within a single imaged *z* slice in the spinal cord of a larva from (A), demonstrating high content imaging and resolution. Scale = 20µm. C) Max projections of longitudinal time lapse two-color overlaid imaging of a single *mnx1*-Gal4;UAS-NTR-mCherry;UAS-GCaMP7 transgenic 72 hpf immobilized larvae at 0, 24, 48, 72, and 96 after immobilization. NTR-mCherry is in red and GCaMP7 is in green. Scale bar = 400 µm. D) 3D reconstruction of larvae at 48 hours after immobilization with corresponding zoom (left) at 0°, 45°, and 90° rotation along the y axis. Scale bar = 400 µm.

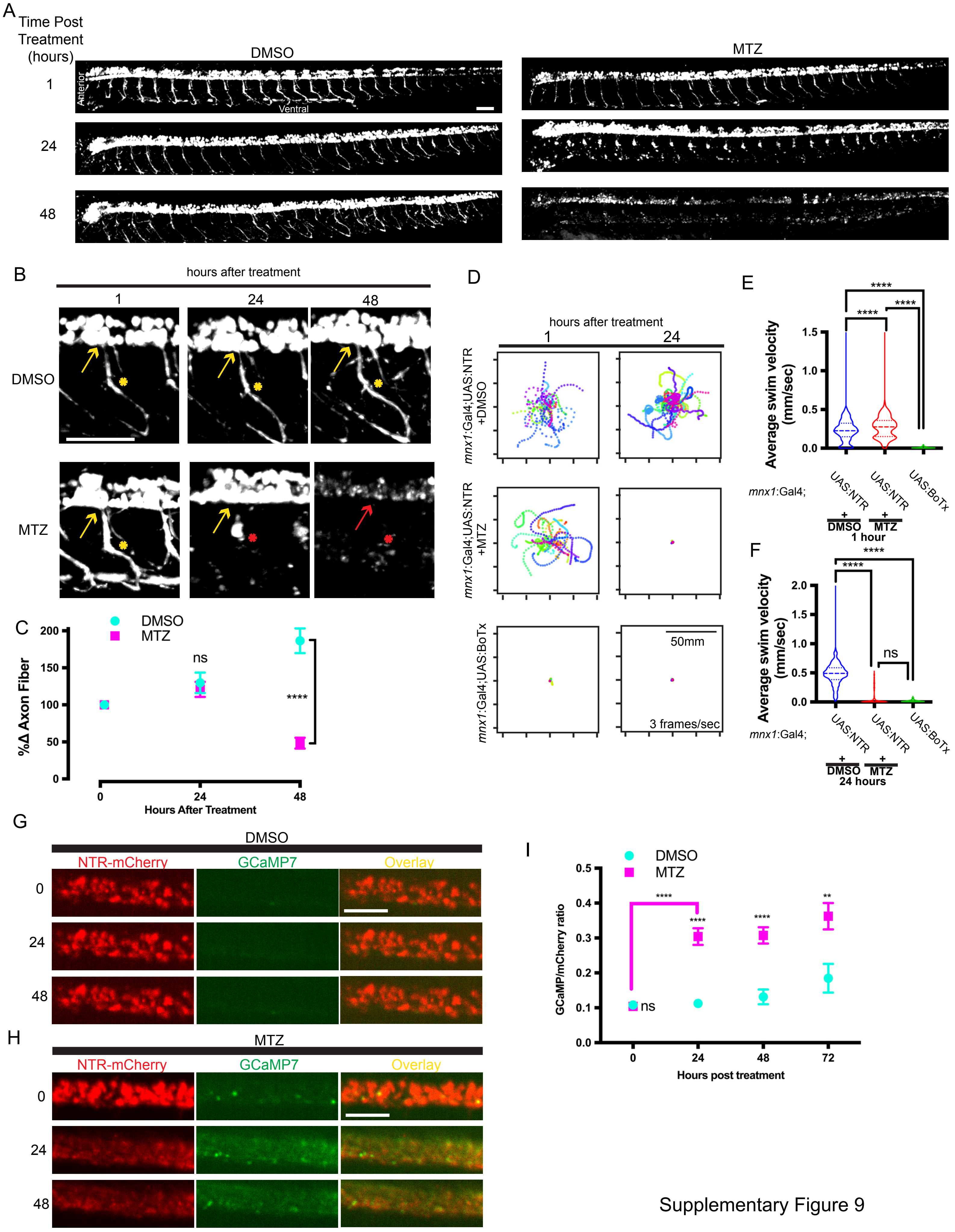

Supplementary Figure 9

Supplementary Figure 9. Acute detection of death in live zebrafish using GCaMP7

A) *in toto* confocal imaging of UAS:EGFP labeled MNs within live, 72 hpf, immobilized zebrafish larvae beginning at 0, 24 or 48 hours after DMSO (left) or 10 μM MTZ exposure (right). Yellow arrow and asterisk indicate developing and intact motor axons and somas respectively, red arrow and asterisk indicate degenerating motor axons, and somas respectively. Scale = 100μm. B) Zoom-in of MNs from (A) exposed to DMSO (top) or MTZ (bottom). Scale = 100μm. C) Quantification of % change of motor axon area per fish after treatment with DMSO (n=7) or MTZ (n=15) (ns, not significant; **** two-tailed T Test $p<0.0001$). D) Traces of touch-evoked swimming behavior from 96hpf larvae after 24 hours of DMSO or 10 μM MTZ treatment. Each fish swimming trace is labeled with a different color and a dot per second. E) Quantification of average velocity of 96hpf larvae touch evoked swimming at 1 hours and 24 hours (n= 358)(F) after DMSO (n= 438) or 10 μM MTZ treatment (n=312), or untreated larvae expressing UAS:BoTx-EGFP in MNs (n=301) (one-sided Kruskal-Wallis test, ns= not significant; **** $p<0.0001$). G) Magnification of MNs from *in toto* confocal imaging of transgenic UAS:NTR-mCherry;UAS:GCaMP7 live immobilized larvae at 0, 24 or 48 hours after DMSO or (H) 10μM MTZ exposure showing increase in GCaMP7 signal in MTZ treated neurons. Scale = 100μm. I) Quantification of mean GCaMP/ mCherry ratio per fish from (F) for DMSO (at 0, 24, 48, 72 hours post treatment n = 6, 6, 5, 2) and MTZ treatments (at 0, 24, 48, 72 hours post treatment n = 9, 8, 8, 7) (ns, not significant; ANOVA Sidak's, **** $p<0.0001$, ** $p<0.01$). Error bars represent SEM.

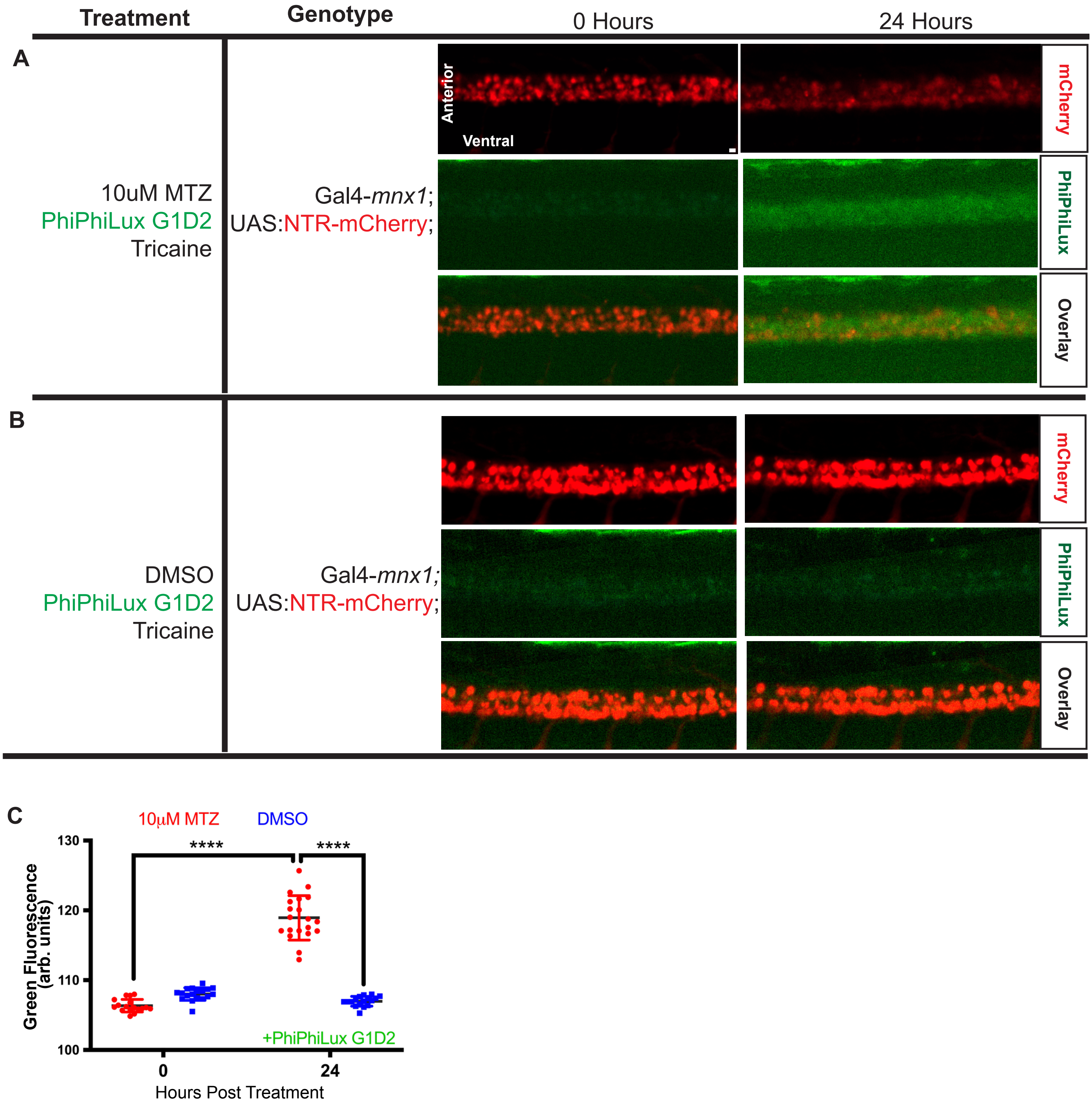
Treatment
Genotype
0 Hours
24 Hours
A
Anterior
Ventral
mCherry
PhiPhiLux
Overlay
10uM MTZ
PhiPhiLux G1D2
Tricaine
Gal4-mnx1;
UAS:NTR-mCherry;
B
DMSO
PhiPhiLux G1D2
Tricaine
Gal4-mnx1;
UAS:NTR-mCherry;
C
10μM MTZ
DMSO
****
****
Green Fluorescence
(arb. units)
130
120
110
100
+PhiPhiLux G1D2
0
24
Hours Post Treatment

Supplementary Figure 10. GCaMP activity correlates with PhiPhiLux signal in NTR-ablated MNs. Characterization of death signal associated with zebrafish larval MNs after 24 hours of chemoablation. A) *mnx1*-Gal4; UAS:NTR-mCherry; show increased PhiPhiLux G1D2 Caspase 3 staining after 24 hours in 10µM MTZ. B) *mnx1*-Gal4; UAS:NTR-mCherry; do not show increased PhiPhiLux G1D2 Caspase 3 staining after 24 hours in DMSO. Scale bar = 10µm. C) Quantification of green fluorescence channel of transgenic *mnx1*-Gal4; UAS:NTR-mCherry larvae exposed to 10µM MTZ (from A) or DMSO (from B) at 0 and 24 hours post treatment (MTZ 0 hours n= 17, 24 hours n=20, DMSO 0 hours n=18, 24 hours n=17). PhiPhiLux stain was applied at 24 hours (ANOVA Tukey multiple comparisons **** denotes $p<0.0001$). Black line represents mean, error bars represent SD.

Supplementary Figure 11

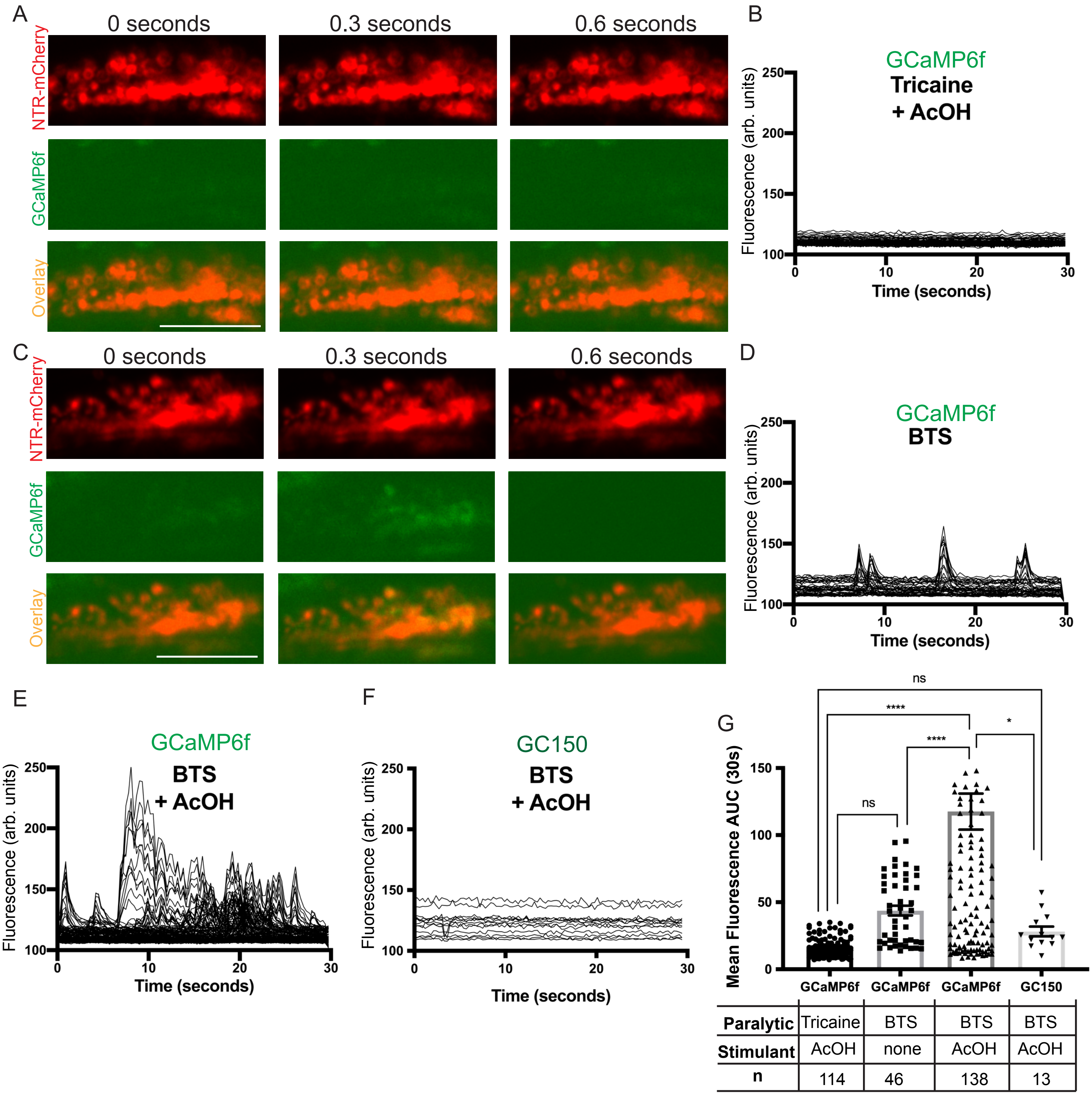

Supplementary Figure 11. Tricaine inhibits activity-related GCaMP activity in zebrafish larvae MNs. A) GCaMP6f fluorescence as an indicator of Ca^{2+} levels over time in zebrafish MNs expressing NTR-mCherry and GCaMP7 immobilized in tricaine. Similar results were found in experiments across at least three different fish. B) Fluorescence traces of GCaMP7 fluorescence over time from MNs when zebrafish is immobilized in tricaine. Scale = 50µm. C) GCaMP6f fluorescence as an indicator of Ca^{2+} levels over time in zebrafish MNs expressing NTR-mCherry and GCaMP7 immobilized in BTS. Scale = 50µm. Similar results were found in experiments across at least three different fish. D) Fluorescence traces of GCaMP7 fluorescence when zebrafish is immobilized in BTS. E) Fluorescence traces of GCaMP7 fluorescence when zebrafish is immobilized in BTS and AcOH is added at 0 seconds. F) Fluorescence traces of GC150 when zebrafish is immobilized in BTS and AcOH is added at 0 seconds. G) Quantification of mean fluorescence by area under the curve of traces of neurons in (B–F). A baseline of the first 5 frames were used as a baseline and peaks less than 50% of the distance from minimum to maximum were ignored. ANOVA Tukey multiple comparisons **** denotes $p<0.0001$, *** denotes $p<0.001$. Error bars represent SEM per neuron, n values represent measurements from individual cells acquired from across at least three independent animals.

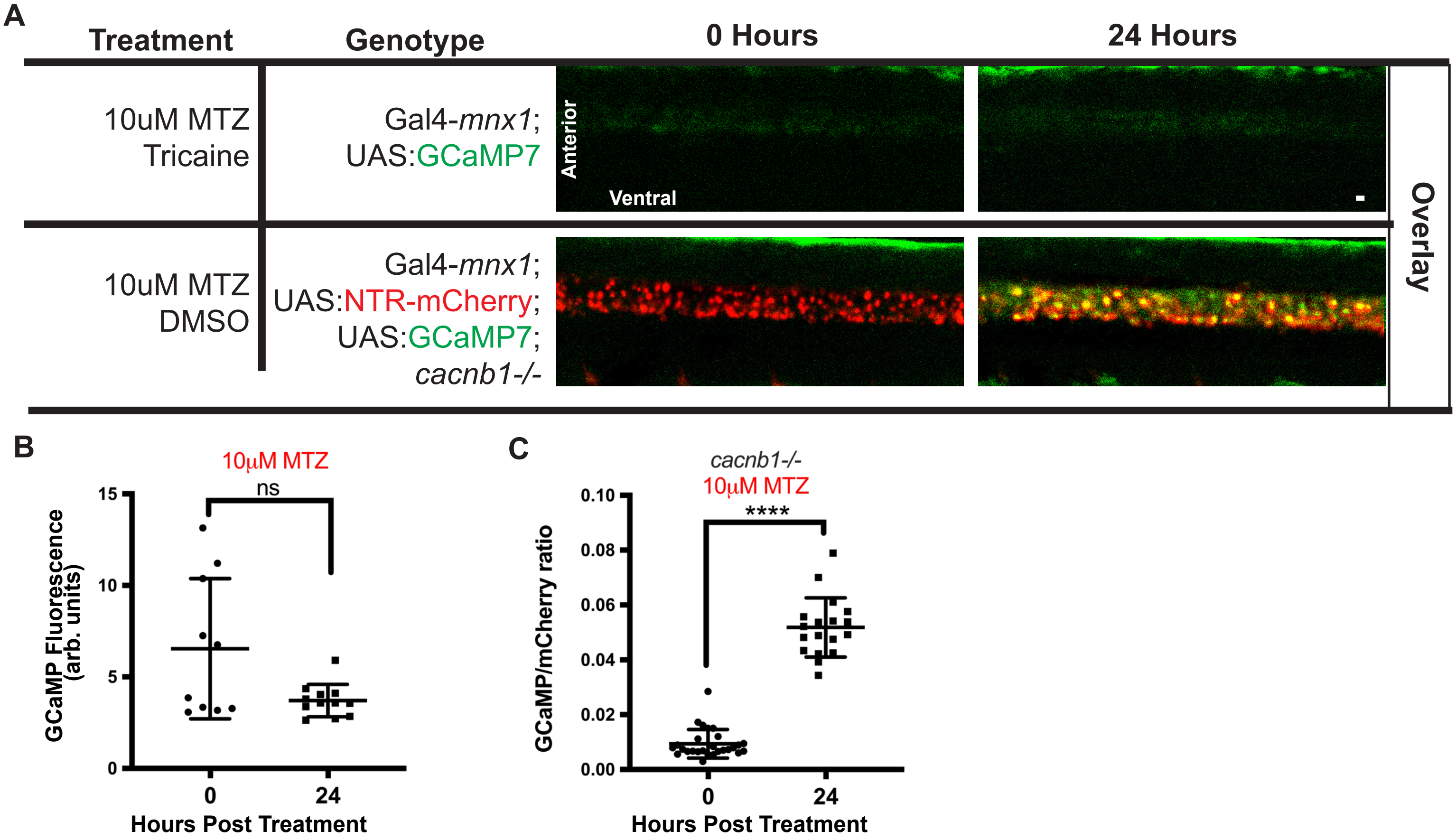
A
Treatment
Genotype
0 Hours
24 Hours
10uM MTZ
Tricaine
Gal4-mnx1;
UAS:GCaMP7
Anterior
Ventral
10uM MTZ
DMSO
Gal4-mnx1;
UAS:NTR-mCherry;
UAS:GCaMP7;
cacnb1-/-
Overlay
B
10μM MTZ
ns
GCaMP Fluorescence (arb. units)
15
10
5
0
0
24
Hours Post Treatment
C
cacnb1-/-
10μM MTZ
****
GCaMP/mCherry ratio
0.10
0.08
0.06
0.04
0.02
0.00
0
24
Hours Post Treatment

Supplementary Figure 12. Death-associated increase in GCaMP signal after MTZ exposure requires NTR expression and is independent of tricaine.

A) (Top) *mnx1*-Gal4;UAS:GCaMP7 larvae exposed to 10µM MTZ do not show increased GCaMP7 signal after 24 hours. (Bottom) Fully immotile *mnx1*-Gal4;UAS:GCaMP7; UAS:NTR-mCherry; *cacnb1-/-* larvae show increased GCaMP7 signal after 24 hours in 10µM MTZ. B) Quantification of GCaMP7 fluorescence in Gal4-*mnx1*;UAS:GCaMP7 larvae at 0 (n= 10) and 24 hours (n=12) post 10µM MTZ treatment (ns = not significant) showing no increase in GCaMP7 signal after exposure to MTZ without NTR expression. Line represents mean, error bars represent SD. Two-tailed Mann-Whitney, ns denotes not significant. C) GCaMP7/mCherry ratio of *mnx1*-Gal4;UAS:GCaMP7; UAS:NTR-mCherry; *cacnb1-/-* larvae at 0 (n=27) and 24 hours (n=18) post 10µM MTZ treatment. Larvae were not exposed to tricaine, two-tailed Mann-Whitney **** denotes $p<0.0001$. Scale bar = 10µm. Line represents mean, error bars represent SD.

Supplementary Table 1. Longitudinal time lapse imaging experiments with rodent primary neurons expressing GEDI

Plate #	# Neurons across timepoints	Co-transfection/Treatment	Mean Time interval (hours)	Time points
**1**	29434	Glutamate	17	13
**2**	93388	Glutamate	20	15
**3**	47070	Glutamate	22	9
**4**	15784	pGW1:HttEx1Q97-mCerulean pGW1:HttEx1Q25-mCerulean	21	8
**5**	52338	pGW1:HttEx1Q97-mCerulean pGW1:HttEx1Q25-mCerulean	24	7
**6**	38744	pGW1:HttEx1Q97-mCerulean pGW1:HttEx1Q25-mCerulean	24	7
**7**	44790	pGW1:HttEx1Q97-mCerulean pGW1:HttEx1Q25-mCerulean	24	7
**8**	46556	pGW1:HttEx1Q97-mCerulean pGW1:HttEx1Q25-mCerulean	24	7
**9**	42374	pGW1:HttEx1Q97-mCerulean pGW1:HttEx1Q25-mCerulean	22	7
**10**	39968	pGW1:HttEx1Q97-mCerulean pGW1:HttEx1Q25-mCerulean	22	7
**11**	24158	pGW1:HttEx1Q97-mCerulean pGW1:HttEx1Q25-mCerulean	22	7
**12**	41522	pGW1:HttEx1Q97-mCerulean pGW1:HttEx1Q25-mCerulean	22	7
**13**	9430	pGW1:HttEx1Q97-mCerulean pGW1:HttEx1Q25-mCerulean	17	6
**14**	7174	pGW1:HttEx1Q97-mCerulean pGW1:HttEx1Q25-mCerulean	24	6
**15**	19744	pGW1:HttEx1Q97-mCerulean pGW1:HttEx1Q25-mCerulean	22	5
**16**	7662	pGW1:HttEx1Q97-mCerulean pGW1:HttEx1Q25-mCerulean	24	6
**17**	9468	pGW1:HttEx1Q97-mCerulean pGW1:HttEx1Q25-mCerulean	24	7
**18**	57000	pGW1:HttEx1Q97-mCerulean pGW1:HttEx1Q25-mCerulean	27	7
**19**	52810	pGW1:HttEx1Q97-mCerulean pGW1:HttEx1Q25-mCerulean	27	7
**20**	44268	pGW1:HttEx1Q97-mCerulean pGW1:HttEx1Q25-mCerulean	24	7
**21**	56146	pGW1:HttEx1Q97-mCerulean pGW1:HttEx1Q25-mCerulean	27	6
**22**	20850	pGW1:HttEx1Q97-mCerulean pGW1:HttEx1Q25-mCerulean	24	7
**23**	15268	pCAGGS:α-synuclein phSyn1:empty	24	7
**24**	14888	phSyn1:TDP43 phSyn1:empty	24	7
**25**	19356	pGW1:HttEx1Q97-mCerulean pGW1:HttEx1Q25-mCerulean	27	5
**26**	17488	pGW1:HttEx1Q97-mCerulean pGW1:HttEx1Q25-mCerulean	24	7

**27**	17300	pGW1:HttEx1Q97-mCerulean<br>pGW1:HttEx1Q25-mCerulean	27	7
**28**	30094	pGW1:HttEx1Q97-mCerulean<br>pGW1:HttEx1Q25-mCerulean	13	15
**29**	58674	pGW1:HttEx1Q97-mCerulean<br>pGW1:HttEx1Q25-mCerulean	22	7
**30**	44426	pGW1:HttEx1Q97-mCerulean<br>pGW1:HttEx1Q25-mCerulean	24	7
**31**	41012	pGW1:HttEx1Q97-mCerulean<br>pGW1:HttEx1Q25-mCerulean	24	7
**32**	49580	pGW1:HttEx1Q97-mCerulean<br>pGW1:HttEx1Q25-mCerulean	24	7
**Totals**	1108764			241

Supplementary Table 2. Zebrafish lines used in study

Figure (s)	Transgenic	Mutation/Background
Figure 6	*neuroD*:GC150-P2a-mApple	roy-/-; nacre-/-; (aka casper)[1]
Figure 6	transient	roy-/-; nacre-/-; (aka casper)[1]
Figure 5 G-I;<br>Supplementary Figure 5<br>Supplementary Figure 7	*mnx:*Gal4[54]; UAS:NTR-mCherry[4];<br>UAS:GCaMP7[2]	Leopard-/-[3];
Figure 5 A-C	*mnx*:Gal4[54]; UAS:mCherry[4];<br>UAS:EGFP	Leopard-/-[3]
Figure 5 D-F	mnx:Gal4[54]; UAS:BoTx-CFP[5]	AB
Figure 5 D-F;<br>Supplementary Figure6	*mnx:*Gal4[54]; UAS:NTR-mCherry[4]	AB
Supplementary Figure 6	*mnx:*Gal4[54];<br>UAS:GCaMP7[2]	Leopard-/-[3]
Supplementary Figure 5<br>Supplementary Figure 6	*mnx:*Gal4[54]; UAS:NTR-mCherry[4];<br>UAS:GCaMP7[2]	cacn1b-/- (aka relaxed, (mi^{90})[6], (ts^{25})[7])
Supplementary Figure 5	1097:Gal4[8]; UAS:EGFP	Leopard-/-[3]

Supplementary Table 3. Expression Constructs used in study.

Construct	Species Expression	Cell Type Expression	Citation
hSyn1:RGEDI-P2a-EGFP	Mammalian	Neuron	This study
pGW1:RGEDI-P2a-EGFP	Mammalian	Ubiquitous	This study
hSyn1:EGFP-P2a-mApple	Mammalian	Neuron	This study
hSyn1:RGEDI-P2a-3xBFP	Mammalian	Neuron	This study
hSyn1:GCaMP6f-P2a-mRuby	Mammalian	Neuron	9
hSyn1:GC150-P2a-mApple	Mammalian	Neuron	This study
hSyn1:RGEDInls-P2a-EGFPnls	Mammalian	Neuron	This study
hSyn1:GC150nls-P2a-mApplenls	Mammalian	Neuron	This study
pGW1:HttEx1Q97-mCerulean	Mammalian	Ubiquitous	10
pGW1:HttEx1Q25-mCerulean	Mammalian	Ubiquitous	10
pCAGGS:α-synuclein	Mammalian	Ubiquitous	11
phSyn1:empty	Mammalian	Neuron	This study
phSyn1:TDP43	Mammalian	Neuron	This study
neuroD: RGEDI-P2a-EGFP-polyA-pDestTol2pA	Zebrafish	Neuron	This study
neuroD: GC150-P2a-mApple-polyA-pDestTol2pA	Zebrafish	Neuron	This study
neuroD: NTR-BFP-polyA-pDestTol2pA	Zebrafish	Neuron	This study
mnx1: GC150-P2a-mApple-polyA-pDestTol2pA	Zebrafish	Motor neuron	This study
mnx1: GCaMP6f-P2a-mRuby-polyA-pDestTol2pA	Zebrafish	Motor neuron	This study

Supplementary References

1. White RM, Sessa A, Burke C, et al. Transparent adult zebrafish as a tool for in vivo transplantation analysis. *Cell Stem Cell.* 2008;2(2):183-189.
2. Muto A, Ohkura M, Kotani T, Higashijima S, Nakai J, Kawakami K. Genetic visualization with an improved GCaMP calcium indicator reveals spatiotemporal activation of the spinal motor neurons in zebrafish. *Proceedings of the National Academy of Sciences of the United States of America.* 2011;108(13):5425-5430.
3. Watanabe M, Iwashita M, Ishii M, et al. Spot pattern of leopard Danio is caused by mutation in the zebrafish connexin41.8 gene. *EMBO Rep.* 2006;7(9):893-897.
4. Nath AK, Ryu JH, Jin YN, et al. PTPMT1 Inhibition Lowers Glucose through Succinate Dehydrogenase Phosphorylation. *Cell reports.* 2015.
5. Sternberg JR, Severi KE, Fidelin K, et al. Optimization of a Neurotoxin to Investigate the Contribution of Excitatory Interneurons to Speed Modulation In Vivo. *Current biology : CB.* 2016;26(17):2319-2328.
6. Zhou W, Saint-Amant L, Hirata H, Cui WW, Sprague SM, Kuwada JY. Non-sense mutations in the dihydropyridine receptor beta1 gene, CACNB1, paralyze zebrafish relaxed mutants. *Cell calcium.* 2006;39(3):227-236.
7. Schredelseker J, Di Biase V, Obermair GJ, et al. The beta 1a subunit is essential for the assembly of dihydropyridine-receptor arrays in skeletal muscle. *Proceedings of the National Academy of Sciences of the United States of America.* 2005;102(47):17219-17224.
8. Scott EK, Mason L, Arrenberg AB, et al. Targeting neural circuitry in zebrafish using GAL4 enhancer trapping. *Nature methods.* 2007;4(4):323-326.

9. Rose T, Jaepel J, Hubener M, Bonhoeffer T. Cell-specific restoration of stimulus preference after monocular deprivation in the visual cortex. *Science (New York, NY).* 2016;352(6291):1319-1322.
10. Arrasate M, Mitra S, Schweitzer ES, Segal MR, Finkbeiner S. Inclusion body formation reduces levels of mutant huntingtin and the risk of neuronal death. *Nature.* 2004;431(7010):805-810.
11. Nakamura K, Nemani VM, Azarbal F, et al. Direct membrane association drives mitochondrial fission by the Parkinson disease-associated protein alpha-synuclein. *The Journal of biological chemistry.* 2011;286(23):20710-20726.
