# Supplementary material for: Genetically encoded cell-death indicators (GEDI) to detect an early irreversible commitment to neurodegeneration
Source: Nat Commun. 2021 Sep 6;12:5284. doi: 10.1038/s41467-021-25549-9 (PMC8421388; doi:10.1038/s41467-021-25549-9)
Supplement: Supplementary file 3 — Reporting Summary [file 41467_2021_25549_MOESM3_ESM.pdf]

nature portfolio

Corresponding author(s): Steve Finkbeiner

Last updated by author(s): Aug 8, 2021

Reporting Summary

Nature Portfolio wishes to improve the reproducibility of the work that we publish. This form provides structure for consistency and transparency in reporting. For further information on Nature Portfolio policies, see our Editorial Policies and the Editorial Policy Checklist.

Statistics

For all statistical analyses, confirm that the following items are present in the figure legend, table legend, main text, or Methods section.

n/a	Confirmed	
☐	☒	The exact sample size (*n*) for each experimental group/condition, given as a discrete number and unit of measurement
☐	☒	A statement on whether measurements were taken from distinct samples or whether the same sample was measured repeatedly
☐	☒	The statistical test(s) used AND whether they are one- or two-sided<br>*Only common tests should be described solely by name; describe more complex techniques in the Methods section.*
☐	☒	A description of all covariates tested
☐	☒	A description of any assumptions or corrections, such as tests of normality and adjustment for multiple comparisons
☐	☒	A full description of the statistical parameters including central tendency (e.g. means) or other basic estimates (e.g. regression coefficient) AND variation (e.g. standard deviation) or associated estimates of uncertainty (e.g. confidence intervals)
☐	☒	For null hypothesis testing, the test statistic (e.g. *F*, *t*, *r*) with confidence intervals, effect sizes, degrees of freedom and *P* value noted<br>*Give P values as exact values whenever suitable.*
☒	☐	For Bayesian analysis, information on the choice of priors and Markov chain Monte Carlo settings
☒	☐	For hierarchical and complex designs, identification of the appropriate level for tests and full reporting of outcomes
☒	☐	Estimates of effect sizes (e.g. Cohen's *d*, Pearson's *r*), indicating how they were calculated

Our web collection on statistics for biologists contains articles on many of the points above.

Software and code

Policy information about availability of computer code

Data collection	Micromanager is an open source platform for programming microscopes. Full automation of the system was performed with Green Button Go (Biosero, Freemont). The code is copyright protected, and its use in performing the described research is patented (U.S. Patent 7,139,415 and U.S. Patent Application 14/737,325). Code is available upon request to the corresponding author. Access to and use of the code is subject to a non-exclusive, revocable, non-transferable, and limited right to use the code for the exclusive purpose of undertaking academic, governmental, or not-forprofit research. Use of the code or any part thereof for commercial or clinical purposes is strictly prohibited in the absence of a Commercial License Agreement from The J. David Gladstone Institutes
Data analysis	Galaxy Project is an open source, web-based platform for computational analysis. https://galaxyproject.org/ Version 16.10 of the platform is used in this work. The infrastructure provided by the Galaxy platform is used to to run our custom code. Custom R and ImageJ scripts were also used in data analysis and their repositories were also used in analysis. No commercial software was used in the analysis.

For manuscripts utilizing custom algorithms or software that are central to the research but not yet described in published literature, software must be made available to editors and reviewers. We strongly encourage code deposition in a community repository (e.g. GitHub). See the Nature Portfolio guidelines for submitting code & software for further information.

Data

Policy information about availability of data

All manuscripts must include a data availability statement. This statement should provide the following information, where applicable:

- Accession codes, unique identifiers, or web links for publicly available datasets
- A description of any restrictions on data availability
- For clinical datasets or third party data, please ensure that the statement adheres to our policy

Raw data acquired by robotic microscopy used in this study are too large to post online but the raw and processed data that support the findings of this study are available from the corresponding author upon reasonable request. Additional representative data, measurements and analysis scripts are available at: https://doi.org/10.5281/zenodo.5107973

Field-specific reporting

Please select the one below that is the best fit for your research. If you are not sure, read the appropriate sections before making your selection.

☒ Life sciences ☐ Behavioural & social sciences ☐ Ecological, evolutionary & environmental sciences

For a reference copy of the document with all sections, see nature.com/documents/nr-reporting-summary-flat.pdf

Life sciences study design

All studies must disclose on these points even when the disclosure is negative.

Sample size	Sample size for survival analysis was determined by the amount of neurons that were transfected in a single batch without any prior assumptions for variance or effect size. Past experience has shown that the high-throughput nature of robotic microscopy provides a 100-1000 fold increase in sensitivity compared to conventional approaches based on single snapshots in time due to the large samples size produced with such an approach. In acute toxin exposure experiments, transfected neurons within the field of view were quantified in each well tested without any prior assumptions for variance or effect size. For zebrafish imaging, sample size was determined by the number of sorted, mounted, and imaged transgenic zebrafish larvae available from a single clutch that were positioned appropriately for quantification (ie in focus and in the field of view) without any prior assumptions for variance or effect size. For transient transfections, cotransfected<br>neurons were located at the first timepoint in all hyperstacks of mounted and imaged fish from a single clutch by their BFP and EGFP or mCherry signal and tracked over time to quantify the change in GEDI signal without any prior assumptions for variance or effect size. For behavioral experiments, an arbitrary amount of videos for quantification were captured at the first time point that had been previously shown to discriminate behavioral defects in zebrafish larvae (Linsley et al PNAS 2017).
Data exclusions	Larvae sometimes died or moved dramatically out of frame over the time-coarse of imaging and behavioral experiments and were censored from analysis.
Replication	At least one other instance of similar phenomenon were observed in each case where described, suggesting these were representative events. All attempts at replication were successful.
Randomization	Zebrafish samples were collected based on availability of experimental conditions within a clutch/imaging experiment and allocated into experimental groups at random. Curation data was acquired based on random sampling of data.
Blinding	Where human curation is present, neuronal death was scored based on the morphology signal without knowledge of the condition or treatment of the cells. TUNEL signal was scored based on whether a cell expressed EGFP or not without looking at RGEDI channel. Investigators were blinded to group allocation during data collection and analysis.

Reporting for specific materials, systems and methods

We require information from authors about some types of materials, experimental systems and methods used in many studies. Here, indicate whether each material, system or method listed is relevant to your study. If you are not sure if a list item applies to your research, read the appropriate section before selecting a response.

Materials & experimental systems

n/a	Involved in the study	
☒	☐	Antibodies
☒	☐	Eukaryotic cell lines
☒	☐	Palaeontology and archaeology
☐	☒	Animals and other organisms
☒	☐	Human research participants
☒	☐	Clinical data
☒	☐	Dual use research of concern

Methods

n/a	Involved in the study	
☒	☐	ChIP-seq
☒	☐	Flow cytometry
☒	☐	MRI-based neuroimaging

Animals and other organisms

Policy information about studies involving animals; ARRIVE guidelines recommended for reporting animal research

Laboratory animals	All animal experiments complied with UCSF regulations and animals are housed in approved facilities with humidity regulated between 30-70%, temperature between 68-79 degrees F, and 12 hour light/dark cycles. Primary mouse (C57BL/6) and rat (Long-Evans) cortical neurons were prepared at embryonic days 20–21 as previously described98. Mouse or rat pups were not sexed, and we expect approximately equal amounts of males<br>and females in our experiments. Zebrafish larvae before 10 days post fertilization were used in this research, and could not be sexed, sp were used without respect to sex.
Wild animals	This study did not use wild animals.
Field-collected samples	This study did not use field-collected animals.
Ethics oversight	Animal studies were approved by UCSF Institutional Animal Care and Use Committee (IACUC) protocols AN183829-02 and AN189188-01.

Note that full information on the approval of the study protocol must also be provided in the manuscript.
